# An improved approach for ecological modeling of social phenomena in Blau space

**DOI:** 10.1371/journal.pone.0289934

**Published:** 2023-08-11

**Authors:** Nicolas L. Harder, Matthew E. Brashears

**Affiliations:** Department of Sociology, University of South Carolina, Columbia, South Carolina, United States of America; The University of Arizona, UNITED STATES

## Abstract

Advances in computation have opened new vistas for modeling of sociodemographic niches and related constructs, enabling us to rectify limitations that unavoidably plagued earlier generations of researchers. This is especially true for Blau space, a sociodemographic niche model used to explore competition between social entities over resources, such as memberships. While this approach has been successful in using probabilistic representations of social networks and resource niches, its modeling framework has remained essentially unchanged for over 40 years, and lacks the ability to make predictions about the future states of sociodemographic space. We present a novel Hybrid Blau space (HBS) model, which utilizes a cellular framework and probabilistic urn models to simulate competition over resources while suffering from fewer limitations. We apply this new model to the General Social Survey, running two sets of models from a series of variables (age, education, income, and sex) and utilize an adjustable range of sociodemographic information for local simulation of organizational competition. We also demonstrate the model’s predictive ability, as well as introduce new methods of validation and fit.

## Introduction

The map is not the territory. To a cartographer, charged with making the enormous manageable, features must be warped in the interests of clarity; mountains look like hills, rivers look like streams, and less impressive features hardly appear. The study of sociodemographic ecologies in general has been plagued by the same difficulties, but confronts the additional complication that while we can physically visit a landmark to confirm what the map tells us, we can rarely do so with a social ecology. For example, traditional Blau space methods (a multidimensional space for modeling population dynamics) have difficulty accounting for categorical and binary variables in a meaningful way. This is analogous to trying to draw an accurate map using a measure of latitude that describes everything as simply “north” or “south”. Similarly, traditional modeling of competition in Blau space focuses on overlap between “niches” (areas of resource exploitation) but fails to adjust for overlaps where few or no resources exist within the ecology. This is akin to a map depicting an island when in fact there is only a small sand bar. Work in the population ecology tradition, another strain of ecological modeling, has proposed mechanisms that could be used to address these issues, including discontinuous and non-polygonal solid (e.g., spheres) niches. But many of these solutions are primarily theoretical in nature without clear implementations or are dependent on specific types of data for their application. No approach yet proposed is easily applicable to many of the problems previously noted in social ecology generally and Blau space models in particular.

We present a framework for improving the maps generated by Blau space and other Sociodemographic niche models [1, 2]. First, we discuss similar approaches from Population Ecology and explain why they fall short of what is needed for modeling Blau space. Then, using this foundation, we introduce the Hybrid Blau Space model (HBS model), a new approach to Blau Space methods (and Sociodemographic niche models by extension) that utilizes a cellular framework inspired by Cellular Automata, an urn model for simulation of influence and competition, and a series of new metrics that describe the behavior of entities (organizations or otherwise) within the ecology. The HBS model advances Blau Space modeling while providing a simple yet versatile framework for implementing the new approach with a variety of datasets. It is also the first Blau Space model that is able to predict future states of the ecology prior to those future states being observed. Finally, we demonstrate the benefits of the HBS model by applying it to the General Social Survey’s Socio-Political Participation module and discuss limitations and future directions for the model.

## Problems with Blau space and sociodemographic niche models

Blau Space is a k-dimensional sociodemographic space for modeling individuals’ associations and behavior in social space. In Blau Space, dimensions represent sociodemographic characteristics that shape social interaction. Examples of these include age, income, or occupational prestige [3–5]. These dimensions allow for the prediction of individuals’ behavior (such as organizational association, influence, and preference adoption) because of the homophily principle, or the tendency for individuals to associate with similar others [4]. Homophily is one of the most robust findings in social science [5, 6] and is observed across a wide variety of characteristics and circumstances in the United States (e.g., [7–9]) and abroad [10].

**Fig 1:**
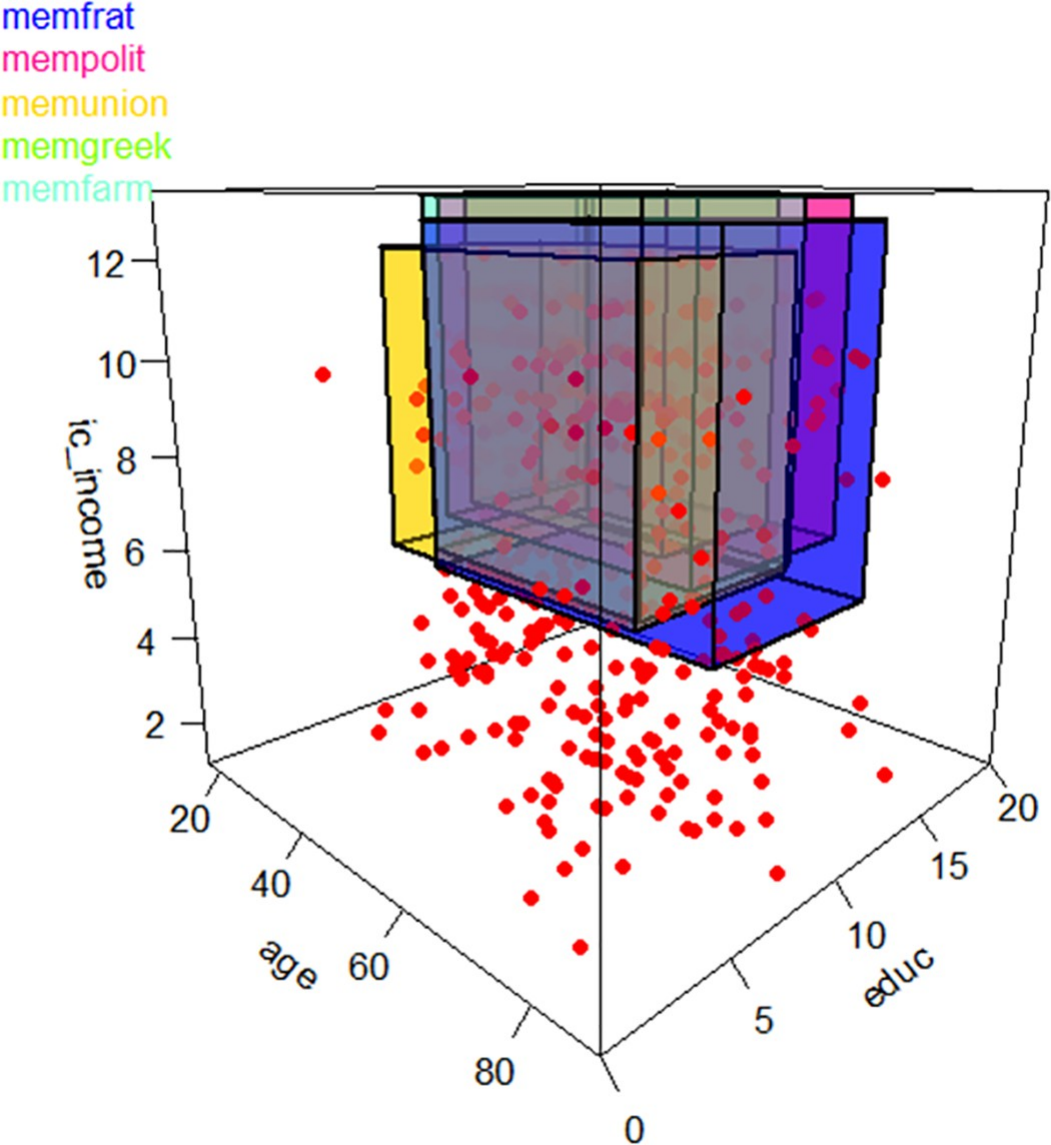
Niche plot using age, education, and income as dimensions.

In traditional Blau Space methods an organization’s niche is computed by starting at the mean value for organization members in each dimension and drawing a boundary at a set range (typically 0.75 standard deviations) above and below the mean. This results in a regular k-dimensional shape (e.g., rectangle or cuboid) 1.5 standard deviations across in each dimension [3, 4] (See Fig 1 in this article for an example).

Traditional Blau Space analysis assumes that competition over resources occurs when, and where, there is overlap between two or more niches, with a greater amount of overlap indicating higher competition for resources [3, 11]. However, in relying solely on overlap, this method of defining competition implicitly assumes that the degree of competition can always be discerned from the level of overlap. When this is not the case overlap is largely meaningless. In addition, traditional Blau Space methods require ordinal or categorical variables be transformed to be continuous (see: [5, 12] for examples).

Fig 1 visualizes a 3-dimensional Blau Space using data from the 1974 General Social Survey (GSS), a common dataset used for testing and validating new Blau Space methods. The figure was created using the R package Blaunet, a precursor to our current modeling effort [11]. This example utilizes the variables age, education in years, and income as Blau Space dimensions and voluntary organizations as the social entities within the space. Memberships are the resource that these organizations seek to exploit. These variables have been used in prior work and have been found informative for predicting organizational membership in prior literature (see: [3, 5] for discussion of the influence of age and education, and [13] for discussion of the influence of income). They are also all continuous variables, allowing them to be utilized in traditional Blau space methods without transformation. Fig 1 includes a subset of organizations from the GSS (Fraternal, Political, Union, Greek, and Farm organizations). This subset matches the organizations included in prior pivotal work on Blau space [5]. It also makes the figure easier to interpret as heavy overlap is observed for all organizations using traditional Blau space methods and if all sixteen organization types (fifteen individual types and an other category) present in the data are included in the figure, the image becomes prohibitively difficult to interpret.

Fig 1 reveals many areas of empty space where individuals (represented as red dots) are not present, but where there is nevertheless overlap of organizational niches. This overlap leads to high competition coefficients, which indicate that organizations are competing over resources with greater intensity, despite the clear lack of resources to compete over. Table 1 shows the competition coefficients between organizational types calculated using the traditional method, based on the spatial overlap between organization niches, rather than overlap in resource demand (see: [3] for original equations and [11] for adaption into an R package). Four of the five organizations exhibit high competition coefficients for multiple other organization types even when we only examine a handful of organization types. If more organization types are included in the model, niche overlap increases and the number of high competition coefficients increases. As a result, traditional Blau space methods can indicate strong competition between organizations for membership regardless of whether any actual competition is present.

**Table 1:** Competition coefficient between subset of voluntary organizations.

	Fraternal	Political	Union	Greek	Farm
Fraternal	0.05	0.85	0.73	0.36	0.9
Political	0.81	0.01	0.77	0.44	0.82
Union	0.81	0.89	0.02	0.27	0.8
Greek	0.79	1	0.54	0	0.83
Farm	0.96	0.9	0.76	0.4	0

Needless to say, since the GSS data are only a sample, there may in fact be individuals present in these areas in the population, but the fraction of the population in these areas should be notably lower. At the same time a high portion of individuals within the ecology are found outside of the organization’s recruitment niches in the lower part of the income distribution. As a result, the model is treating all areas as equally rich in resources, even though they clearly are not. Consequently, competition coefficients can be quite high even if resources (i.e., individuals to recruit for membership) are not available in the locations of overlap [14].

### The population ecology approach

The main issues identified in traditional Blau space methods are the assumption that an organization’s niche is a regular polygon of uniform size, and that all areas within the niche are of equal importance. Using traditional Blau space methods as demonstrated in Fig 1, we see that this assumption results in niches that are heavily overlapping, which should indicate competition. However, as shown in Table 1, because competition is calculated from niche overlap and not shared resource demand, prior methods do not accurately identify membership recruitment competition and, by extension, organizational behavior. This is in part because of the assumptions traditional Blau space methods place on niche shape, forcing a regular cellular shape on what is unlikely to be a uniform recruitment space.

In comparison to Blau space, population ecology models have explored other methods of shaping ecologies and niches, such as composing niches as a series of spheres instead of rectangles or cuboids [15, 16]. However, although these methods of modeling are possible, they often require complex solutions to overcome newly introduced issues (for example, utilizing “deep holes” and algorithms for close packing to overcome unusable space where spheres are not able to touch; see: [15] for more information).

Population ecology has also introduced the idea of discontinuous or irregular niches, which partition resources into at least two sections along a dimension [17]. However, this is typically implemented on theoretical data, and not in practice with empirically obtained datasets. The continuous population level approach is still frequently applied in empirical analyses even when assumptions ought to be relaxed (see: [18] for an example using generalized estimating equations (GEE)). Put differently, researchers often settle for what may be a substantially inaccurate approximation because it is easier and not because it is appropriate. More effort has been given to better empirical approaches to categorical data, either using the categorical variables for grouping resources or as dimensions [2, 19], but without decisive success. For example, [19] utilize only categorical variables as dimensions in their ecological model, transforming variables that would generally be continuous (i.e., age) into categories via a cutoff value. However, crossings between these categorical dimensions are summarized using Herfindahl indices and then averaged, creating a distribution ([19], pg. 419). For some variables this transformation allows for the preservation of variation because the distribution of values naturally have a high and low point (i.e., age and education have increasing values and a starting point), but for other variables this is not the case. For example, religious and political preference are used as categories, and if these categories are collapsed then meaningful differences between levels are lost.

Lastly, there is a distinction between how Blau space and population ecology models capture organizational behavior and engagement. In population ecology the appeal of an organization in segments of the ecology is an important element of the theory. The extent to which the organization pursues these resources (i.e., its engagement) is defined by the offerings that an organization provides for individuals [1, 2]. In other words, the focus is on the actions of the organization to secure resources.

The focus of theories that utilize Blau space is the gathering of resources from members or consumers in the ecology. However, the emphasis is on individuals’ behavior within the ecology instead of the agentic behavior of organizations within the ecology. The resource emphasized by Blau space theories is time, not monetary capacity, making individual investment more important than organizational engagement [3, 4]. Organizations tend to attract individuals who are similar to their existing members because that is who they have access to. At the same time individuals have a disposition to associate with those similar to themselves, resulting in social forces that structure interactions and generate shared social and cultural understanding [20]. As such, Blau space approaches do not require that we consider organizations as agents in the first place.

## The Hybrid Blau space model

Blau space is a useful framework for modeling social influence [9, 11], preference adoption [21, 22], and membership selection and recruitment processes [3, 23]. However, there are several issues with Blau space modeling that either limit its application or make interpretation of the space difficult and misleading. These include difficulty in interpreting overlapping niches and problems incorporating non-continuous variables (such as categorical or binary) as dimensions of the space. In addition, the existing Blau space modeling framework, as well as many applications, are largely descriptive and lack the predictive ability to forecast change in the ecology (see: [5] for a partial exception). Therefore, we propose a new model, the Hybrid Blau space model (HBS model), that grants heightened flexibility in the range of variables that can be implemented and allows for prediction of future states of a social ecology.

The HBS model is a complete revision of traditional Blau space methods that is implemented in the R coding environment. The HBS model makes two fundamental changes to the underlying implementation of Blau space. The first is a reconceptualization of Blau space’s regular polygonal niches in a continuous space into irregular niches in a space composed of discrete ordered cells. The second is the utilization of a probabilistic Urn Model to simulate competition between entities in the space over a series of cells. This method is analogous to the micro-step updates used in other agent-based models (e.g., SAOM [24], or RSiena [25]). Further advancing Blau space modeling, we have included several scalable parameters to fit the model to the specific data being analyzed and adapted a convergence criterion to the model in order to test the fit of simulated model output. The inclusion of a convergence criterion is directly related to the simulation-based nature of our model and its ability to forecast future states of the ecology. The HBS model not only captures processes in Blau space, but also utilizes prior events to make predictions and compare them to a distribution of possible outcomes, again similar to existing network inference models (i.e., RSiena or ERGMs). Below we explain each of these model changes and additions in more detail and the implications these revisions have for interpreting Blau space.

First, we reconceptualize Blau space as composed of a series of discrete cells defined by crossings of values for each sociodemographic variable included in the model. Cells can be defined using equal intervals (i.e., all intervals are equal across the range of the dimension) or can be scaled in accordance with the social distinction entailed by specific sociodemographic dimension value differences (e.g., [6]). This change from a continuous to a discrete cellular structure allows for continuous, ordinal, and binary variables to be utilized in our models more easily. It is conceptually simpler to discretize a continuous variable than to map an ordinal or categorical variable onto a continuous dimension, therefore this approach requires fewer assumptions about the nature of the data. In this framework niches are no longer limited to regular and continuous polygons, but instead are a series of individual cells from which organizations draw members, and thus the niche can take on any of a variety of highly irregular shapes.

Individuals who belong to the same cell are within an interval distance of each other, with the interval determined by levels of each dimension, while those individuals in different cells differ in at least one dimension by at least one interval. For example, a cell in the space would exist for the crossing between 32 years of age and a college education, and anyone with matching values on those dimensions would populate this cell in the ecology. This means that social distance in the Hybrid model is represented by the number of cells that separate groupings of individuals.

The second change in the HBS model is the incorporation of a probabilistic urn model, a class of stochastic models used for discrete outcomes [26]. These models rely on a metaphor such that discrete outcomes are imagined as balls of different colors that are added to an urn. Assuming mixing, a random draw from this urn will yield a ball of a given color with a probability proportional to its relative frequency in the urn. In our case, each membership is a unique “color” of ball and memberships are contributed to a common urn by several cells (see discussion below). Updating memberships in a cell then utilizes this urn, thereby capturing the social influence exerted by organizational members who are proximate in Blau space. This allows the model to simulate influence in a localized area of Blau space by considering the influence pressure of surrounding cells, with different organizations contributing balls (memberships) to a common urn as a function of their recruitment success. Moreover, it can account for differing levels of competitive pressure in each cluster of cells, avoiding the need to assume a uniform distribution of resources.

The urn model takes advantage of the cellular structure and consists of three phases. The first phase begins by selecting a cell at random, referred to as a focal cell, and identifies the surrounding cells. These cells are referred to as “neighbors” of the focal cell and are viewed as those that have an influence on the focal cell. This area of influence is referred to as the cell neighborhood and is scalable by the researcher. In brief, membership in the focal cell is assumed to depend on the distribution of memberships it contains, as well as those in neighboring cells. For the focal cell and neighboring cells this is the total count of each resource (e.g., possible memberships) and the total count of consumed resources (e.g., held memberships).

In the second phase the model determines the relative prevalence of each organization within the cell neighborhood by calculating each organization’s proportion of total memberships in a cell neighborhood (e.g., count of memberships in an organization divided by the count of all memberships in any organization). These proportions are then used as the probability that an individual will be recruited to, or leave, an organization.

In the third and final phase the probability of memberships are multiplied by the number of persons in the focal cell, reassigning all memberships in the focal cell according to the relative prevalence of an organization in the surrounding cells. During the final phase, after the process has been completed for the selected focal cell, a new focal cell is selected from the ecology randomly (with replacement). This process continues until a researcher set convergence criterion is satisfied.

### Scaling in the cell neighborhood

In addition to changing the modeling of Blau space to a discretized cellular structure, we have introduced the ability to scale the cell neighborhood as desired. This allows for a larger cell neighborhood, or area of influence on the focal cell, to be considered during the simulation of competition (analogous to adjusting the size of a traditional niche, but locally instead of globally). Individuals in neighboring cells, even if they are not directly adjacent to a focal cell, may still be influenced via local ties or passing observation [13]. For example, a 26 year old is likely to have friends close to their age (e.g., a range of 1–5 years), but may not have friends the same age as them. Regardless, these individuals would still influence their voluntary organization membership adoption. Because the model simulates recruitment and influence in disconnected niches, the scaling of a cell neighborhood during micro-steps of the probabilistic urn model is disconnected from prior steps, thus the model still functions as a first-order Markov Chain.

**Fig 2:**
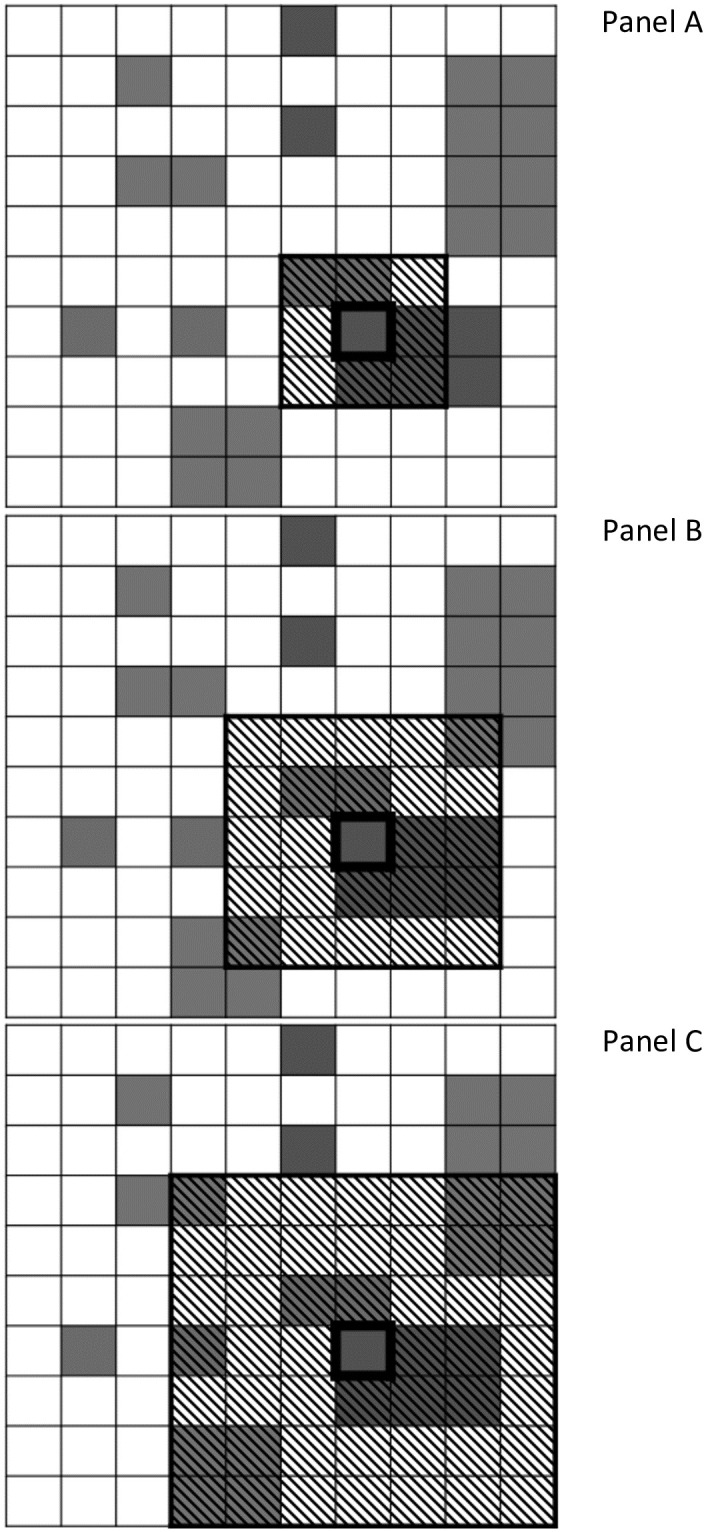
Illustration of cell neighborhood scaling.

Scaling of the cell neighborhood is handled as a parameter set by the researcher during model setup. This parameter sets the radius of cells around the focal cell that comprise the focal cell’s neighborhood. Fig 2 shows an illustration of a scaling process for selecting the cell neighborhood using a two-dimensional ecology. The model selects a focal cell (thick black bordered cell) at random from the ecology and then considers cells within *x* intervals from the focal cell to be within the cell neighborhood (horizontally shaded area), and will utilize this area when calculating relative prevalence of an organization type’s membership. For the models considered in this paper *x* ranges from 1 to 3. For Fig 2, which utilizes a 2-dimensional ecology as an example of this process, this results in cell neighborhoods of 9, 25, and 49 cells. The results we present later in this paper utilize 3 dimensions, meaning that the cell neighborhood includes a total of either 27, 125, or 343 cells.

### Dimensionality, niche shape, and social distance

With our choice of modeling approach also come assumptions about the dimensionality, possible niche shapes, and the meaning of distance in the model and how it relates to social distance. The HBS model differs from conventional Blau space methods in its implementation of niches within the ecology. In conventional Blau space methods niches are forced to be continuous and result in rectangles or cuboids that are bound by a range (typically 1.5 Std. Dev) from the mean value an organization type has on a dimension. In contrast, the HBS model allows for discretized niches that are formed of any combination of cells within the ecology. Niches are composed of a series of smaller rectangles or cuboids that represent neighborhoods within the ecology.

Although dimensionality can be scaled up or down by the researcher (with a minimum of 2 dimensions and a practical maximum based on interpretation limits and computation time) the cellular approach forces some assumptions on the HBS model. Because the ecology is shaped into a series of discretized cells instead of a continuous space, we can rely on previous work on modeling networks with Cellular Automata that shows how changes in levels on a dimension (i.e., increasing or decreasing discretization), and changes in the number of dimensions included, will change social space (see: [15, 27]). Although this is a limitation to our modeling approach, it results in predictable model behavior as an increase in dimensions under a rectangular or cuboid structure also increases the neighboring cells [28]. This means that additional dimensions increase the chance that an individual is in a cell with neighbors, as well as increase the number of total cells in the ecology resulting in increased social distance as individuals can now differ on more dimensions. In addition, a discretized cellular space also avoids complications introduced by efforts to use other shapes, such as spheres.

### Convergence criterion and method

Convergence of the HBS model is assessed by comparing the average number of memberships an organization holds within each cell of the ecology and the number of memberships an organization holds in each cell after a set series of micro-steps. As the model runs it saves out the results of every *n* number of permutations as a snapshot of the ecology. The number of permutations is similar in practice to the number of micro-steps in an RSiena model [25]. These snapshots are used to generate an average of memberships an organization holds in a cell across a convergence range, or a researcher specified number of previous permutations of the ecology. This average is then compared to the latest simulated ecology to check for changes to the cell counts for each organization. If the number of cells that are different from the last comparison of the ecology to the convergence average is equal to or lower than a specified threshold (models in this paper use a threshold of 3), then the model is considered to have converged. This process is repeated for each of the organizations in the model, and if all organizations meet the threshold the model is considered to have converged. However, if any single organization fails to meet the convergence threshold, then the model continues running for another set of permutations. The goal is to permute the space to a point where ongoing changes to cells in the ecology have receded into a sort of stochastic background noise, rather than showing evidence of continued large-scale change.

In practice each organization’s memberships are represented in an independent array, meaning there are n arrays matching the number of organizations in the ecology, with each array mirroring the other in terms of dimensions. Model convergence is done at the cell level, comparing the cells at the same coordinates in each array to see if additional meaningful changes can be made in the space. Although comparison is done on an organization-by-organization basis (via arrays) this does not contribute to the method beyond being a computational convenience.

Once the model reaches convergence for a set of micro-steps, the entire process is repeated from the initial data for a researcher specified number of global iterations. For our analyses this value is set at 125, meaning that we simulate outcomes from identical starting conditions 125 times until each reaches convergence as specified above. We also tested a global iteration value of 25, which yielded comparable results. We use 125 global iterations here to be conservative. Results for each global iteration are saved out and the means are used for computing a series of evaluation metrics. These metrics will ultimately be used to identify a central tendency across all global iterations to characterize the ecology’s likely fate, as well as to characterize the variability in this fate.

### Extensiveness, cell focused intensiveness, and range focused intensiveness

Because the underlying mechanisms of Blau space modeling change in the HBS model, a new series of metrics are required to analyze and evaluate the resulting behavior of organizations within the space. These metrics are: Extensiveness, Cell Focused Intensiveness (CFI), and Range Focused Intensiveness (RFI). They were developed to summarize the position and extent of our niche equivalents, and thus are somewhat akin to the niche centers and widths in traditional Blau space. Extensiveness, CFI, and RFI, are used as evaluative metrics to assess organization recruitment behavior in the ecology (i.e., describe their size, spread, and concentration).

#### Extensiveness

Extensiveness is a measure of the range or breadth of an organization’s recruitment area in cellular Blau space. It is defined (Eq 1) as the count of cells in a regular polygon, with its limits being the upper and lower values of the organization in each dimension. In Eq 1, O_i_ is the count of the number of cells between the minimum and maximum values of organization members in each dimension, therefore describing a regular polygon from which the organization is ostensibly recruiting members. This value is then normalized to the unit interval by dividing the number of cells encompassed by an organization’s polygon (O_i_), by the number of cells in a similar polygon defined by the maximum and minimum values for individuals in all organizations in the ecology (R). The result is the fraction of the space that is currently occupied by any organization that is also occupied by organization i. In our current implementation, this fraction is calculated for the initial observation, and averaged across each model run that attains local convergence. Extensiveness is obviously most analogous to conventional niche breadth calculations, but as such is relatively insensitive to the irregular niches that our approach permits.


$$Extensiveness=\frac{O_{i}}{R}$$ (1)

#### Intensiveness

Intensiveness is a measure of success in organizational recruitment at specific locations within the ecology. To understand success at both the local, cell-based level, as well as the global, ecology-based level, we utilize two different forms of Intensiveness: Cell Focused Intensiveness (CFI) and Range Focused Intensiveness (RFI).

**Cell Focused Intensiveness** (CFI) is a measure of success in monopolizing resources in a localized area (i.e., cell) within the larger ecology (Eq 2). The number of members an organization holds in a cell (x_ijk_) is divided by the number of individuals within the cell (c_ijk_). This proportion is calculated for each cell in which an organization holds members, and then summed together and divided by the total number of cells from which an organization derives members (Q). CFI ranges from 0 to 1, in which a value of 0 means that the organization holds no members in any cell in the ecology and a value of 1 means that the organization holds all possible members in each cell in which they hold members. This means that CFI increases as organizations have more success in recruitment within specific locations in the ecology, and in ecologies where there is a great deal of competition over members, we would expect CFI to be lower for all organizations. Critically, however, an organization may have a high CFI even if it is located only in a few cells because the metric tracks recruitment success in cells where the organization is present, rather than its ability to blanket the space.


$$CFI=\frac{\sum \left(\frac{X_{ijk}}{C_{jk}}\right)}{Q}$$ (2)

**Range Focused Intensiveness** (RFI) is calculated similarly to Cell Focused Intensiveness, with the exception that the denominator (O_i_) is the number of cells within the range, or regular polygon, from which an organization recruits’ members (Eq 3). This changes the interpretation of intensiveness from the success of recruitment in a series of specific areas within the ecology, to the success of recruitment within the range from which an organization recruits’ members. Again, this value ranges from 0 to 1, in which 0 indicates that the organization holds no cells in the ecology, and 1 indicates that it holds members in each cell in its range and holds all members in those cells.


$$RFI=\frac{\sum \left(\frac{X_{ijk}}{C_{jk}}\right)}{O_{i}}$$ (3)

CFI and RFI are meant to jointly provide insight into the success of an organization’s recruitment strategy. CFI measures the average success organizations have in gaining and holding memberships in each individual cell where they hold membership. This means the metric is only measuring behavior in locations where the organization already holds members, ignoring cells where it is not competing for resources. On the other hand, RFI considers the entire organization’s range of recruitment, and so measures its success in appealing broadly. CFI and RFI don’t have to scale together. For example, an organization could have a high CFI because it holds all or near all the memberships in 4 cells at corners of the ecology and at the same time a very low RFI because of it’s wide recruitment range. Likewise, an organization could hold few memberships in cells right next to each other, resulting in a low CFI but a high RFI because the recruitment range is very short. Thus, in a sense, CFI captures the depth of an organization’s competitive success whereas RFI captures the breadth.

## Data

Data for this study comes from the General Social Survey (GSS), a regular probability sample of non-institutionalized US adults. We rely on the demographics provided by the GSS, and the module on Socio-Political Participation, which includes information on voluntary social organization membership. Data from the 1974 to 2004 collection waves of the GSS are utilized, all of which include the Socio-Political Participation module. In later years of the GSS (1988–1994, and 2004) the module was only provided to a subsample of survey participants [29]. Because the number of complete entries and the number of participants in the GSS vary by year, the number of final observations for each year also varies from 882 to 1,108.

The Socio-Political Participation module only collected membership in organization types, not specific organizations. Social organizations are therefore aggregated by organization type (e.g., sports organizations, literature organizations) in the data, and thus we analyze organizational “species” rather than individual organizations, as has been customary in prior research [3, 5]. We assume that organizations within these type classifications share similar goals, structures, and institutional logics, including recruitment and competition behaviors [2].

The GSS does not provide a count of the number of discrete organizations included in each organization type (i.e.: Fraternal, Service, Veterans, Political, Union, Sport, Youth, School, Hobby, Greek, National, Literature, Professional, and Church), or the number of organizations within each type that an individual belongs to (i.e., we may know they belong to at least one sporting organization but cannot discriminate between individuals belonging to one or more than one of the same type of organization). Therefore, we measure membership in 16 different types of voluntary social organizations, and individual respondents may be members of as few as none of them, or all sixteen. Obviously, it would be preferable to have data on individual organizations, but ecological research typically focuses on the species level rather than the individual level. Much as biological ecologists use a sample of individual members of a species to characterize overall inter-species competition, we use a sample of individual memberships to characterize inter-organization type competition.

We employ sociodemographics that have been reported as dimensions in other Blau space papers, specifically age [3, 5], education [5], income [13], and sex [12]. When possible, we do not recode the underlying variables. In other words, an individual who is 18 years old and has a high school degree has a value of 18 in age and a value of 12 in years of education. Age ranges from 18 to 89 years, with a mean of 42.41 and a standard deviation of 16.21 for all waves of data. Age is technically truncated because the highest age category is “89 or older”. However, because there are few observations in this part of the age distribution (26 individuals, making them a little less than 1/500^th^ of the sample included in our analysis) it is unlikely that this truncation has a meaningful effect. Education ranges from 0 to 20 years, with a mean of 12.99 and a standard deviation of 3 for all waves of data. Sex is a binary variable measured by self-report, where a 1 is coded for female and a 2 is coded for male.

Income is taken from the family income variable provided by the GSS and is adjusted for inflation on a year-by-year basis. This is done by mid-point scoring (e.g., [30]: pg. 203–204) each category of the income variable by year and then adjusting this value for inflation with the reference point being 2004. These new categories are then discretized into a set of 12 categories to match the original number of categories included in the GSS.

### Dataset fit and ecology resources

The HBS model imposes three requirements on a dataset: sociodemographics that can be discretized, a resource or set of resources of interest, and social entities that are competing over those resources. Ideally, resources would be measured in terms of both memberships and behaviors, allowing researchers to model the actual level of investment an individual has in organizations of which they are a member. However, very few datasets collect this information, and so the ideal is rarely attainable in practice. As such we, like other researchers in the Blau space tradition, rely on self-indicated membership. We know from prior research that organizations’ access to resources stems from the organization’s members themselves [3, 31]. In return for resources from members, organizations provide a range of benefits to individuals who join the organization (e.g., social belonging, networking, social and economic capital). Individuals also vary in the amount of resources that they can provide to organizations, thus limiting the number of organizations that they can be a member of and the level of investment they are able to provide to each organization that they are a member of. Even with the perfect dataset we still would lack an accurate measure of individual capacity unless something like time use data was collected. However, we are able to approximate the membership and investment capacity by looking at the total number of memberships an individual holds across all organization types. We do not explore resource capacity in this paper but find this line of work important and hope to explore it in future work.

## Models and results

### Models

We fit four models using 4 different sociodemographics as dimensions (age, education, income, and sex) and varied the number of cells included in the cell neighborhood (1–3). We ran our models with two different dimension sets, one set that included age, education, and income as dimensions, and a second set that included age, education, and sex. These crossings result in 4 models, model 1—Age, Education, Income with a range of 1 (AEI1), model 2—Age, Education, Income with a range of 2 (AEI2), model 3—Age, Education, Income with a range of 3 (AEI3), and model 4—Age, Education, Sex with a range of 1 (AES1). Because the scaling of the local simulation range is equal on all dimensions, the model including sex (a binary variable) is only run with a range of 1. Table 2 outlines the 4 models that result from crossings between dimensions included and neighborhood ranges. Each model utilizes the same parameters, a limit of 40,000 permutations to reach convergence, and a total of 125 global iterations. If the model reaches 40,000 permutations and has not converged the model resets, ensuring that if a model fails to satisfy the convergence criterion that it does not run indefinitely.

**Table 2:** Included models.

	Age, Education, Income	Age, Education, Sex
Range 1	Model 1—AEI1	Model 4—AES1
Range 2	Model 2—AEI2	
Range 3	Model 3—AEI3	

### Sign analysis and successful trend prediction

The goal of the HBS model is to predict trends in organizational size and behavior, as measured by Extensiveness and Cell and Range Focused Intensiveness. Because the population size for the ecology is different between years, the data are cross-sectional (the same individuals are not surveyed in each year), and the magnitude of an effect between years can change because of both population size and exogenous effects, it is more appropriate to focus on trends than point predictions. We utilize sign analysis, allowing us to track and predict trends in organizational behavior between GSS data collection waves. We compare observed time periods and the results simulated from them, checking if the signs of each pairing match. When the signs match for a majority of the included organizations, indicated by 75% of signs matching for a comparison between simulation and observed, we consider the model successful in predicting trends. This comparison is repeated for each metric as it is possible for the model to succeed in predicting the trend behavior of one metric while failing to predict the trend of another. Because the metrics we have developed for the HBS model capture different aspects of organizational recruitment behavior, we see this as an advantage rather than a defect in that it imposes a more stringent test and yields more informative results. It should be noted that there are many possible effects that could influence the behavior of social entities, and that many of these are presently exogenous to the model. As such, exact predictions are not yet realistic. However, the homophily principle, on which Blau space approaches are based, is one of the most robust findings in all of social science [20]. As such, the model should capture the bulk of the ecological effects that influence the behavior of social entities.

**Fig 3:**
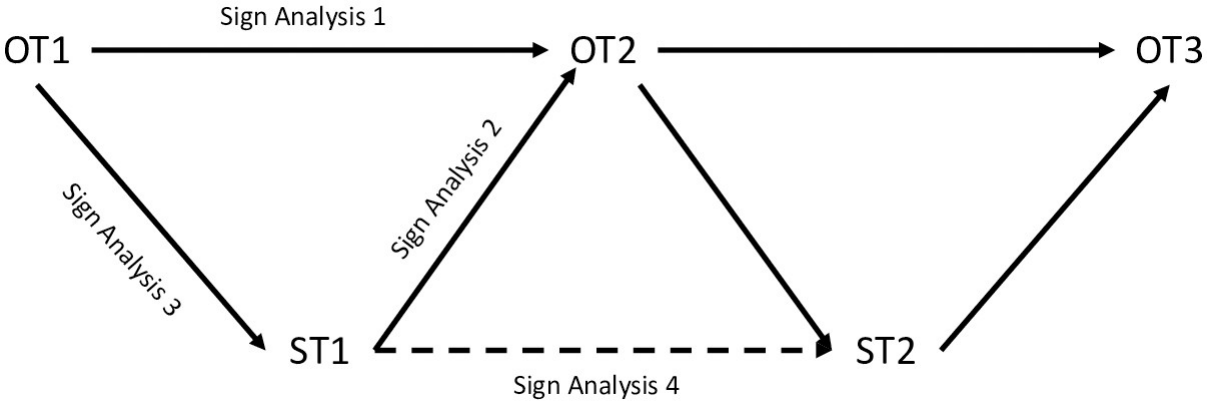
Sign analysis diagram.

Fig 3 shows all sign analysis comparisons between observed data collections and simulated observations. Sign analysis 1 compares two observed time periods (labeled as OT1 and OT2). This allows us to determine whether two or more observation periods correlate. Sign analysis 2 compares a simulated time period (ST1) to a future observed time period (OT2), thus indicating if the simulated time period is correlated with the future observed time period and if the model is simulating change in the direction of the observed change (e.g., whether a predicted increase and an observed increase match).

Sign analysis 3 compares a previously observed time period (OT1) to a simulated time period (ST1). Sign analysis 3 is useful as an indicator of how closely the future simulated time point matches the original data used to generate it. Ideally these two observations would be correlated.

Interpretation of sign analysis 2 and sign analysis 3 can be challenging because it is notoriously difficult to link simulation time to clock time, and this issue can be exacerbated by exogenous factors that may influence the behavior of both organization types and individuals. For example, political turmoil might drive individuals from leisure-based organization types (e.g., sports organizations and book clubs) to politically oriented ones (e.g., nationalist or political organizations), but this is an exogenous effect that cannot be predicted from organizational competition. Given that exogenous factors will likely prolong the time needed to reach equilibrium, and that these factors are absent from the model, it is likely that observed processes play out over longer periods of time than the simulation captures. For sign analysis 2 this will likely not make a substantial impact, as correlation with sign analysis 1 will indicate that the simulation is trending in the direction dictated by observed organizational competition. But this issue is likely to be especially meaningful for sign analysis 3 as differences between sign analysis 1 and sign analysis 3 are driven relatively more by exogenous forces and less by the overall trend. Sign analysis 2 does not suffer from this issue to the same extent and should be able to capture whether the model is predicting change in the correct direction.

Mismatches in sign analysis 3 could indicate that the model is a poor fit, but could also indicate that the model is simulating a temporal result that is prior to the second observation (i.e., on the way to the current observation) or would have been obtained by the second observation in the absence of exogenous factors. Additionally, over permutation of the model or excessive model fitting may improve sign analysis 3, while simultaneously compromising the sign comparisons that are useful for trend prediction, sign analysis 1 and 2.

Lastly, sign analysis 4 compares two simulated time periods (ST1 and ST2). This comparison mirrors the purpose of sign analysis 1, but for simulated time periods instead of observed. These four sign analyses are repeated for all pairings of observed time periods and simulated results.

Table 3 lists the average number of times different crossings of sign analysis trend together as a percentage. We also provide the average percentage of trend prediction successes for sign analysis crossings across all years included in S1 Appendix. Looking first at Extensiveness, we find that any comparison for the baseline model (AEI1) that excludes sign analysis 3 has acceptable predictive ability, with predictive ability decreasing slightly as cell neighborhood increases using the same dimensions (AEI2 and AEI 3). Looking at model AES1, which includes sex as a dimension, we see that the model again succeeds in trend prediction for Extensiveness, with the exclusion of any comparison that includes sign analysis 3. For Cell and Range Intensiveness, model AEI1 generally has predictive success (again when sign analysis 3 is excluded), but we see a steep decline as the cell neighborhood is increased. The exception to this is the model AES1, which generally has trend prediction below the threshold for both Cell and Range Intensiveness. Lastly, trends in population (counts of memberships per organization type) are again predicted most successfully for model AES1, with decreased trend prediction in the AEI2 and AEI3 models, and generally poor trend prediction in the AES1 model. We focus on the AEI1 model, the model best at accurately predicting trends, to discuss model prediction insights.

**Table 3:** Sign analysis summary averages.

	Model 1—AEI1					
	S1-S2	S2-S3	S1-S3	S2-S4	S1-S4	S3-S4
Extensiveness	0.9910	0.5223	0.5313	0.9911	0.9821	0.5134
Cell Focused Intensiveness	0.8527	0.4777	0.6250	0.8929	0.8616	0.5670
Range Focused Intensiveness	0.9152	0.4777	0.5625	0.9152	0.9375	0.5446
Population	0.9598	0.4821	0.5089	0.9599	0.9732	0.5223
	Model 2—AEI2					
	S1-S2	S2-S3	S1-S3	S2-S4	S1-S4	S3-S4
Extensiveness	0.9554	0.5491	0.5893	0.9732	0.9375	0.5580
Cell Focused Intensiveness	0.7902	0.3527	0.5357	0.7455	0.6964	0.4464
Range Focused Intensiveness	0.7679	0.3259	0.5580	0.7768	0.8214	0.4955
Population	0.8661	0.3839	0.5045	0.8795	0.9286	0.4955
	Model 3—AEI3					
	S1-S2	S2-S3	S1-S3	S2-S4	S1-S4	S3-S4
Extensiveness	0.9286	0.5625	0.6295	0.9330	0.9196	0.6027
Cell Focused Intensiveness	0.6741	0.2098	0.5268	0.7009	0.7054	0.4643
Range Focused Intensiveness	0.7321	0.2857	0.5536	0.6875	0.7500	0.5000
Population	0.8482	0.3527	0.4911	0.8571	0.8929	0.4955
	Model 4—AES1					
	S1-S2	S2-S3	S1-S3	S2-S4	S1-S4	S3-S4
Extensiveness	0.8125	0.4107	0.5089	0.8661	0.8214	0.4643
Cell Focused Intensiveness	0.6741	0.3304	0.5670	0.6161	0.6384	0.4732
Range Focused Intensiveness	0.6295	0.2143	0.5134	0.6652	0.5714	0.3884
Population	0.7232	0.3304	0.5223	0.7411	0.8036	0.5179

### Trend prediction insights

Now that we have a sense of the quality of model fit, what does the model tell us concretely about how organization types are behaving in the ecology? In contrast to existing Blau space methods, that express change only in terms of niche movement (e.g., [5]) we are able to provide a more complete picture of how competition impacts an organization type’s success in recruiting and retaining members. To match the examples provided in the niche plots above (Fig 1) we will focus on the same 5 organization types (Fraternal, Political, Union, Greek, and Farm Organizations). This choice simplifies comparison of our results to prior work. Because our illustration includes 14 year by year comparisons of GSS data (years 1974–2004) there are many possible comparisons, but our goal is to show examples of how the HBS model can be used. Thus, we focus only on the early years of the GSS; specifically, the waves collected in 1974, 1975, 1977, 1978, and 1980. For transparency, Table 4 still provides all 14 year by year comparisons. Because our primary interest is the change from one time period to another, we will compare Sign Analysis 1 and Sign Analysis 2, and when the two signs match, discuss the resulting consensus outcome.

**Table 4:** Trend predictions for select organizations.

	Model 1—AEI1									
	Sign 1					Sign 2				
1974–1975	Fraternal	Political	Union	Greek	Farm	Fraternal	Political	Union	Greek	Farm
Extensiveness	-	+	+	+	+	-	+	+	+	+
Cell Intensiveness	+	-	+	+	+	+	-	+	+	+
Range Intensiveness	-	-	+	+	+	+	-	+	+	+
Population	-	-	+	-	-	-	+	+	-	+
1975–1977	Fraternal	Political	Union	Greek	Farm	Fraternal	Political	Union	Greek	Farm
Extensiveness	+	-	+	-	+	+	-	+	-	+
Cell Intensiveness	-	+	-	-	-	+	+	-	-	-
Range Intensiveness	-	+	-	+	-	+	+	-	+	-
Population	-	-	-	+	-	+	-	-	+	-
1977–1978	Fraternal	Political	Union	Greek	Farm	Fraternal	Political	Union	Greek	Farm
Extensiveness	-	+	-	+	-	-	+	-	+	-
Cell Intensiveness	-	-	-	-	-	+	-	-	-	-
Range Intensiveness	+	-	-	-	+	+	-	-	-	-
Population	+	-	-	-	-	+	-	-	-	-
1978–1980	Fraternal	Political	Union	Greek	Farm	Fraternal	Political	Union	Greek	Farm
Extensiveness	+	+	-	+	+	+	+	-	+	-
Cell Intensiveness	+	+	+	+	+	+	+	+	-	+
Range Intensiveness	+	+	+	+	+	+	+	+	+	+
Population	-	-	-	+	+	-	+	-	+	-
1980–1983	Fraternal	Political	Union	Greek	Farm	Fraternal	Political	Union	Greek	Farm
Extensiveness	-	-	+	+	-	-	-	+	+	+
Cell Intensiveness	-	-	-	-	-	-	-	-	-	-
Range Intensiveness	-	-	-	-	-	-	-	-	-	-
Population	+	+	+	+	+	+	+	+	+	+
1983–1984	Fraternal	Political	Union	Greek	Farm	Fraternal	Political	Union	Greek	Farm
Extensiveness	+	+	+	+	+	+	+	+	+	+
Cell Intensiveness	-	+	+	-	+	-	+	-	-	-
Range Intensiveness	-	-	+	-	+	-	-	+	-	+
Population	-	-	+	+	-	+	-	+	+	+
1984–1986	Fraternal	Political	Union	Greek	Farm	Fraternal	Political	Union	Greek	Farm
Extensiveness	+	-	-	-	-	+	-	-	-	-
Cell Intensiveness	+	+	+	+	-	+	+	+	-	+
Range Intensiveness	-	+	-	+	-	-	+	-	+	-
Population	-	-	-	-	-	+	-	-	-	-
1986–1987	Fraternal	Political	Union	Greek	Farm	Fraternal	Political	Union	Greek	Farm
Extensiveness	+	+	-	-	-	+	+	-	-	-
Cell Intensiveness	+	-	+	+	+	+	-	+	+	+
Range Intensiveness	+	-	+	+	+	+	-	+	+	+
Population	+	+	+	+	+	+	+	+	+	+
1987–1988	Fraternal	Political	Union	Greek	Farm	Fraternal	Political	Union	Greek	Farm
Extensiveness	-	-	-	-	-	-	-	-	-	-
Cell Intensiveness	-	+	-	-	+	+	+	+	+	+
Range Intensiveness	-	+	-	-	-	+	+	-	-	-
Population	-	-	-	-	-	-	-	-	-	-
1988–1990	Fraternal	Political	Union	Greek	Farm	Fraternal	Political	Union	Greek	Farm
Extensiveness	+	-	+	+	-	+	-	+	+	+
Cell Intensiveness	-	+	-	-	+	-	+	-	-	-
Range Intensiveness	-	+	-	-	+	-	+	-	-	-
Population	-	-	-	-	-	-	-	-	-	-
1990–1991	Fraternal	Political	Union	Greek	Farm	Fraternal	Political	Union	Greek	Farm
Extensiveness	+	-	+	+	+	+	-	+	+	+
Cell Intensiveness	+	-	-	+	-	+	-	-	+	-
Range Intensiveness	+	-	-	+	-	+	-	-	+	-
Population	+	+	+	+	+	+	+	+	+	+
1991–1993	Fraternal	Political	Union	Greek	Farm	Fraternal	Political	Union	Greek	Farm
Extensiveness	+	+	-	+	-	+	+	-	+	-
Cell Intensiveness	-	+	-	-	-	-	+	+	-	+
Range Intensiveness	-	+	+	-	+	+	+	+	-	+
Population	+	-	+	+	+	+	-	+	+	+
1993–1994	Fraternal	Political	Union	Greek	Farm	Fraternal	Political	Union	Greek	Farm
Extensiveness	-	-	-	-	-	-	-	-	-	-
Cell Intensiveness	+	-	+	+	+	+	-	+	+	+
Range Intensiveness	+	+	-	-	-	+	+	-	-	-
Population	-	-	-	-	-	-	-	-	-	-
1994–2004	Fraternal	Political	Union	Greek	Farm	Fraternal	Political	Union	Greek	Farm
Extensiveness	+	+	+	+	+	+	+	+	+	+
Cell Intensiveness	-	+	-	-	-	+	+	-	-	-
Range Intensiveness	+	+	+	+	+	+	+	+	+	+
Population	+	+	+	+	+	+	+	+	+	+

Starting with the 1974 to 1975 transition period, we see 4 out of the 5 focal organization types increase their Extensiveness, with only Fraternal organizations experiencing a decrease in Extensiveness. However, this does not match up with membership or Cell and Range Intensiveness. Greek organizations decrease in membership but see an increase in their success in holding individuals in specific cells and across the ecology (Cell and Range Intensiveness). A similar mismatch is seen with Fraternal organizations, which experience a decrease in Extensiveness and membership count but an increase in Cell Intensiveness. In the case of both the Fraternal and Greek organizations this can be interpreted as the organization types losing membership overall, but retaining a set of dedicated members in areas where they are already present. Nevertheless, while the overall interpretation is similar, each organization goes about this in different ways, with Fraternal organizations decreasing their Extensiveness and thus having a smaller but more concentrated recruitment area, and Greek organizations solidifying their existing members both in individual cells and across the ecology while also retaining their recruitment area. In other words, each of these organization types are increasing their recruitment behavior in areas of the ecology where they have had the best prior success.

In the 1975 to 1977 transition, we observe several trends reverse. For example, Fraternal organizations see an increase in their Extensiveness and Greek organizations see a decrease in their Extensiveness. At the same time Greek organizations experience an increase in Range Intensiveness and membership count. However, most organization types lose memberships in this year. This could be the result of prior recruitment strategies or exogenous factors not accounted for in the model (such as an economic recession or changes in norms around volunteering behavior). Looking forward to the 1977 to 1978 transition we observe a continuation of this trend, with the only notable change being that many of the Cell and Range Intensiveness trends switch from positive to negative, and a general decrease in membership count is observed in all but Fraternal organizations.

Lastly, looking at the 1978 to 1980 transition we see a strong trend within the ecology for organization types to increase their Extensiveness and Cell and Range Focused Intensiveness. This trend does not transfer to an increase in memberships, again with only Greek organizations seeing an increase in their membership count. Although we end our discussion of trends here, if continued to the next transition period (1980 to 1983) we do see an increase in membership count across all these focal organizations.

Although this is a brief interpretation of part of the model output, it demonstrates that the benefit of our model is not only that it is able to provide predictions for the next observed time period, but also that the model can be used to track behavioral trends of organization types over time. We can capture nuanced information about how organizational recruitment and retention are changing, by extension pointing to quite different developmental trajectories. Given the increasing prevalence of algorithm-driven micro-targeting, the ability of the model to capture highly local competitive dynamics in a series of nuanced metrics offers manifold opportunities to researchers.

### Goodness of fit tests

To further validate our model, we assess its goodness-of-fit (GoF) to the data. Our GoF test relies on the standard inverted logic of a classic t-test, with a high p-value indicating that the observed data and simulated data are indistinguishable from each other (i.e., could both be derived from the same population). Here the samples are the observed and simulated descriptive metrics used to summarize organizational behavior in the ecology; if metrics generated from the simulated data are statistically indistinguishable from the metrics generated from the observed data, we may conclude that the model yields a good fit to the data.

We compare the single observed metric to a population of simulated results that are generated from our model runs. If the t-test for this comparison has a p-value above 0.75 then we consider the model to yield a good fit to the data. This comparison is made for each organization type within the model, meaning a total of n (in this case n = 15) t-tests are executed on a different set of metrics. In other words, a series of simulated outcomes for the ecology are used to create a distribution that should contain the observed ecology within a confidence interval if the model fits well. The cutoff p-value of 0.75 is arbitrary, as the goal here is to set a p-value that is significantly large in order to be certain that the model is a good fit. P-values larger than 0.75 could be used for the cutoff, but as the criteria for model GoF increases, small artifacts in the distribution of model outcomes are more likely to create false negatives. At this time a 0.75 threshold is appropriately stringent but may be raised as model development continues.

**Fig 4:**
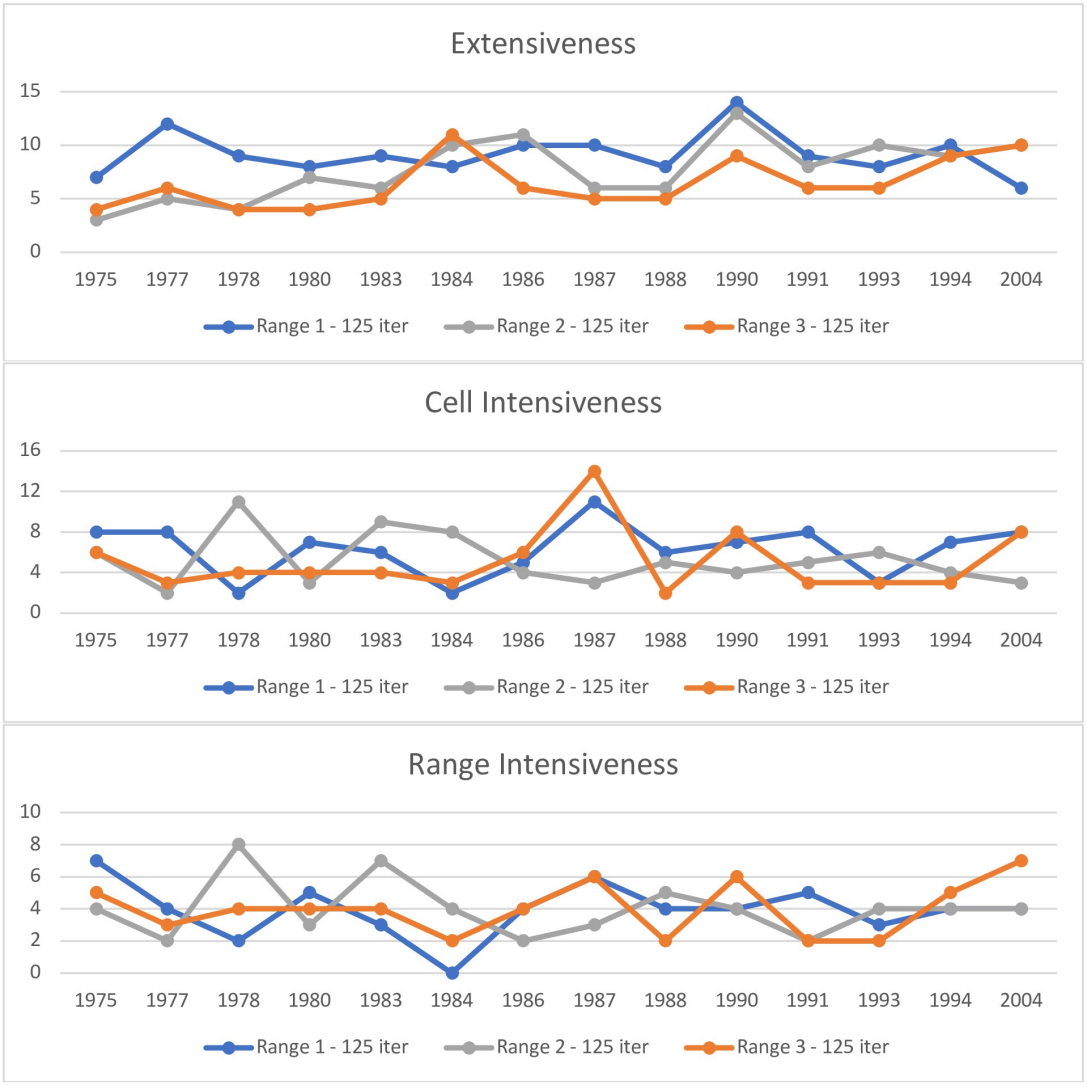
Count of organizations with a sufficient GoF by descriptive metric^a^. ^a^Only AEI models shown in this figure.

**Fig 5:**
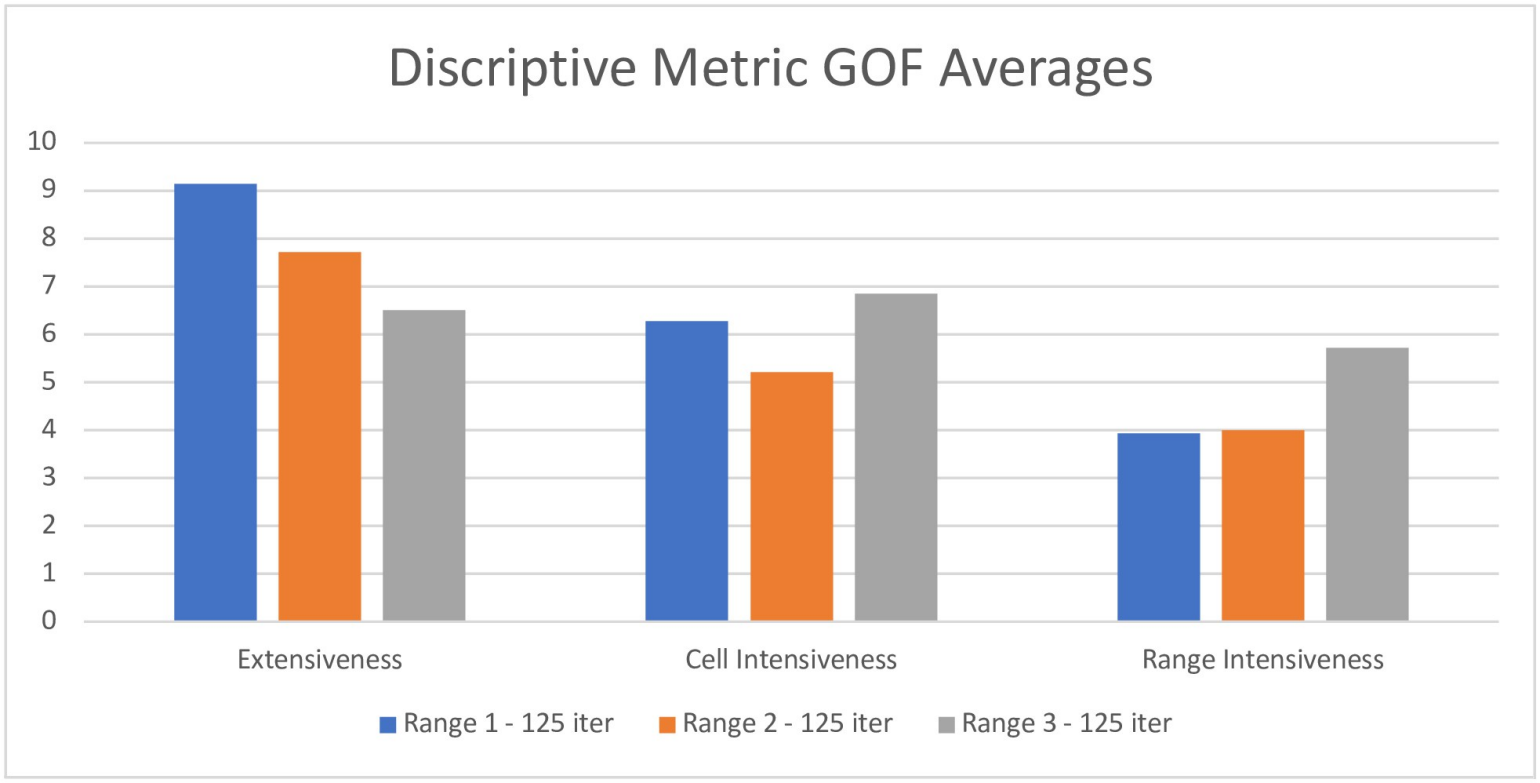
Average number of times the GoF threshold is reached across years in model^a^. ^a^Only AEI models shown in this figure.

The descriptive metrics of Extensiveness, Cell Intensiveness, and Range Intensiveness are evaluated using our GoF test. Fig 4 shows line graphs of the count of organization types whose t-test have a p-value above 0.75 within a given year. Fig 5 shows a bar graph of the average number of times that the 0.75 p-value threshold was reached for each metric across all years. Given the large number of tests, these figures are the most straightforward way to examine the results.

Looking at the average GoF across all years model by model we see that GoF varies by size of the cell neighborhood. For Extensiveness, model AEI1 has the highest number of good fitting p-values. For models AEI2 and AEI3 we see a slow decline in average GoF for Extensiveness (falling to 7.7 and 6.5 respectably). However, for Cell Focused Intensiveness and Range Focused Intensiveness we actually see model AEI3 demonstrating a better GoF on average. Surprisingly, we see model AEI1 preform the worst for Range Focused Intensiveness, dropping to an average of 3.9 across all years. However, this is likely because the increase in cell neighborhood ranges did not scale evenly with the social meaningfulness of different categories on that dimension. In other words, for some dimensions this made the fit to data considerably worse as individuals at these social distances really do not exert an influence on membership behavior.

When considering model AES1, the GoF for Extensiveness is 14.14 on average, while Cell and Range Focused Intensiveness are much lower on average (3.64 and 2.57 respectively). This model only utilizes a range of 1, but that range encompasses a large portion of the ecology because the sex variable is binary, reflecting the substantial theoretical implications of methodological choices in ecological modeling. Although not implemented in this project, the next update to the HBS model will be able to utilize cell neighborhoods that are not perfect cuboid shapes (i.e., range on each dimension does not need to be the same). It is also possible to include more than 3 dimensions in the model. Although this will increase the chance that individuals within a cell have neighbors, it also increases the total cell count of the model, adding more possible neighboring locations to the ecology. Both aspects should be tuned with guidance from the theory a researcher is using, the variables available as dimensions, and existing knowledge about those dimensions, much as one is guided by similar factors in fitting conventional models.

## Discussion

This paper demonstrates the advances offered by our new HBS model, specifically addressing three shortcomings of traditional Blau space modeling. First, traditional modeling methods both indicate competition where there are no resources and fail to include large swathes of resources in the niches, resulting in misleading conclusions about an organization type’s behavior in the ecology. This means that estimated competition over resources in the ecology is simply wrong, and often is dramatically incorrect. Second, traditional methods limit the variety of dimensions that can be included, resulting in a poor representation of social space. Specifically, categorical and binary variables are not possible to include without transformation that can remove important information from the variable or add information that was not originally present. Third, traditional Blau space models are able to illustrate competition between social entities over resources but are not able to predict future states of a social ecology.

The HBS model is our response to the drawbacks of existing approaches and includes a discretized cellular space that allows for a wide range of variables to be included, a probabilistic urn model that simulates competition and influence in social space, a series of descriptive metrics to illustrate the behavior of organizations within the space, and a GOF metric to measure model fit to observed data. We believe the HBS model is a practical model for three reasons. First, although there are advanced population ecology models that already incorporate a number of the innovations we propose (e.g., [2]), these models are often not actually used for, or suited to, practical modeling of sociodemographic niche theories. Second, although other population ecology models have applied innovations, like irregular niches and disconnected niches [17], these innovations are applied theoretically instead of as practical additions to a modeling framework. In addition, the innovations often require a complex solution to work around problems introduced by the “solutions” themselves. Our model demonstrates practical applications for irregular niches and disconnected niches by utilizing available real-world data to simulate organizational recruitment behavior and make predictions and does so in a way that does not introduce exotic additional drawbacks.

In addition to these benefits, our simulation approach provides a predictive model for sociodemographic niche theories. Specifically, we provide simulation of year-by-year microevolutionary changes in organization behavior instead of overarching macroevolutionary changes in behavior. We believe that our focus on microevolutionary changes (i.e., at the cell level) is more useful then macroevolutionary changes (i.e., at the niche level) because it allows researchers to look closely at specific regions of the ecology and aspects of interest. It can also be aggregated to a more macro approach when desired. This approach acknowledges and builds on existing theory of ecological behavior that points to an evolutionary trend taking place over a long period of time and being composed of small changes in behavior; these macro changes over long periods of time are the drivers of actual changes of entity behavior within the ecology ([32]; see [33] for discussion of macroevolutionary vs. microevolutionary trends).

Our model does have limitations, as do most models utilized in population ecology. For example, many of the actual resources entities need in the ecology (i.e., memberships for organizations) are endogenous to the model itself. Yet, there are outside forces that impact recruitment that are not captured by competition. However, this is a limitation that has befallen all prior population ecology models and is typically the result of difficultly in collecting information to measure such aspects of the ecology. In addition, there are a number of processes that either we are unable to control for in the model or have not controlled for (such as the effect of membership recruitment and retention from past years or larger global trends). It is possible future versions of the model will be able to include these factors, but at present they remain exogenous. Although membership in an organizational type and total number of organizations a member is a part of are provided (and can be used to indicate level of investment), the GSS does not collect measures of behavioral investment that would be of interest to Blau space modeling. Examples include time investment in an organization per week and fiscal donations to an organization. Future work should take into account not only self-reported membership in organizations, but also an individuals’ level of investment in an organization, in order to more accurately measure the amount of resources an individual is providing, and the capacity and individual has for participation. If the data were available, however, the HBS model is presently capable of taking advantage of it.

Lastly, this application of the HBS model, as with most applications of sociodemographic ecological models, utilizes organization types instead of individual organizations (i.e., we predict what organization types an individual will join, but not membership in individual organizations). There is no theoretical or modeling reason that prevents us from utilizing individual organizations, and we have done so in earlier work developing the HBS model (see: [14]). However, an organization that falls into an organization type should conform to a template that makes it similar to a range of other organizations. Likewise, organizations of the same type confront similar ecological challenges both in the formal marketplace (i.e., business organizations) and in the time economy (e.g., voluntary associations). It should therefore be possible to sensibly abstract to the level of organization types, and parallels biological ecology which models behavior at the level of species [34].

Future work utilizing this model should attempt to gather and utilize such information, as the current state of the HBS model would allow for it to be used in the simulation process, but simplifications and assumptions must be made. For example, we weight the individuals in our model to allow them to be members of more than one organization at a time. However, we have no way of measuring the actual time and energy an individual is able to contribute to voluntary organization membership or how much time and energy a given membership requires, and so cannot easily specify how these limits will prevent them from joining multiple organizations.

In the HBS model we have tried to keep the parameters versatile, compartmentalized, and simple enough to be easily learned by a neophyte. In doing so we hope to set an example for future social ecological models, thereby encouraging adoption of these methods. We aim to continue development of the model and release a companion to existing modeling software for sociodemographic niches. In summary, the HBS model is a replacement for traditional Blau space methods when simulating competition and attempting to predict an organization’s recruitment behavior. In its current state the model shows promise and has several useful features for modeling theories that fall under the umbrella of sociodemographic niche theories.

## Supporting information

S1 Appendix Sign analysis counts by year and model final. (XLSX)
